# Identification of *potential* opinion leaders in child health promotion in Sweden using network analysis

**DOI:** 10.1186/1756-0500-5-424

**Published:** 2012-08-08

**Authors:** Karin Guldbrandsson, Monica K Nordvik, Sven Bremberg

**Affiliations:** 1 Division of Social Medicine, Department of Public Health Sciences, Karolinska Institutet, Stockholm, Sweden; 2 The Swedish National Institute of Public Health, Östersund, Sweden; 3 Department of Social Work, Mid-Sweden University, Östersund, Sweden

**Keywords:** Network analysis, Opinion leaders, Child health promotion

## Abstract

**Background:**

Opinion leaders are often local individuals with high credibility who can influence other people. Robust effects using opinion leaders in diffusing innovations have been shown in several randomized controlled trials, for example regarding sexually transmitted infections (STI), human immunodeficiency virus (HIV) prevention, mammography rates and caesarean birth delivery rates. In a Cochrane review 2010 it was concluded that the use of opinion leaders can successfully promote evidence-based practice. Thus, using opinion leaders within the public health sector might be one means to speed up the dissemination of health promoting and disease preventing innovations. Social network analysis has been used to trace and map networks, with focus on relationships and positions, in widely spread arenas and topics. The purpose of this study was to use social network analysis in order to identify potential opinion leaders at the arena of child health promotion in Sweden.

**Results:**

By using snowball technique a short e-mail question was spread in up to five links, starting from seven initially invited persons. This inquiry resulted in a network consisting of 153 individuals. The most often mentioned actors were researchers, public health officials and paediatricians, or a combination of these professions. Four single individuals were mentioned by five to seven other persons in the network. These individuals obviously possess qualities that make other professionals within the public health sector listen to and trust them.

**Conclusions:**

Social network analysis seemed to be a useful method to identify influential persons with high credibility, i.e. *potential* opinion leaders, at the arena of child health promotion in Sweden. If genuine opinion leaders could be identified directed measures can be carried out in order to spread new and relevant knowledge. This may facilitate for public health actors at the local, regional and national level to more rapidly progress innovations into everyday practice. However, effectiveness studies of opinion leaders in the public health sector still have to be performed.

## Background

A prerequisite for getting a new method into everyday practice, which often takes a long time [1], is that potential users are aware of the method. According to Everett Rogers, who developed the theory “Diffusion of Innovations” about the spreading of new ideas, opinion leaders are individuals who can influence other people [2]. Rogers has described opinion leaders with the following characteristics: they have greater exposure to mass media, greater contact with change agents, greater social participation and higher socioeconomic status compared to their “followers”. Further, they are by Rogers described as more cosmopolite and more innovative. Dearing describes opinion leaders as local, influential persons with high credibility [3] and Flodgren et al. describe them as people who are seen as likeable, trustworthy and educationally influential [4]. According to Grimshaw et al. the informal leadership of opinion leaders is not a function of position or status, but earned by competence, social accessibility and conformity to the systems norms [5].

Robust effects using opinion leaders in diffusing innovations have been shown in several randomized controlled trials, for example regarding STI and HIV prevention [6], mammography rates [7] and caesarean birth delivery rates [8]. Doumit et al. concluded in a Cochrane review 2007 that “the use of opinion leaders can successfully promote evidence-based practice” [9]. This finding was confirmed by an updated version of the review, with six additional trials, by Flodgren et al. [4]. Thus, using opinion leaders within the public health sector might be one means to speed up the dissemination of new health promoting and disease preventing methods.

Rogers suggests four methods to identify opinion leaders: the socio-metric method, the informant method (by Grimshaw et al. labelled the key informant method [10]), the self-designating method and observation [2]. Positive correlations among these four measures were obtained and they were all by Rogers assumed to be about equally valid. In a recent Cochrane review by Flodgren et al. the socio-metric method and the informant method were assessed to be equally valid [4]. Valente and Pumpuang have reviewed ten techniques used to identify opinion leaders [11]. One of these techniques is the snowball method, where individuals are asked to nominate considered opinion leaders in the community in a repeated process. According to Valente and Pumpuang the snowball method is one of the most likely to identify actors to whom others ask for advice [11]. There are, to our knowledge, no studies on opinion leaders in the arena of child health promotion in Sweden.

Social network analysis has been used to trace and map networks in widely spread arenas and topics, such as infections [12], terrorism [13], suicide [14], social marginalisation [15], happiness [16] and information exchange [17]. The main characteristic of social network analysis is the focus on relationships and positions in social networks, which facilitates a deeper understanding of on-going processes in social structures [18].

The purpose of the study described in this paper was to investigate if social network analysis was useful in identifying *potential* opinion leaders at the national arena of child health promotion in Sweden.

## Methods

A feasibility study using social network analysis was designed. The informant and snowball methods [2,4,11] was used in order to trace potential opinion leaders. The informant method implies asking subjectively selected key informants in a system to designate opinion leaders, simply by asking them who they assess to be “leaders” in the system, and the snowball method implies that this was done in a repeated process.

Thus, in order to identify influential persons with high credibility in the field of child health promotion, seven leading actors, mainly policy makers, at the national level within the Swedish public health sector, such as the Children's Ombudsman, were contacted by e-mail (see Table 1). The seven start actors were, in a short message, asked to give names and e-mail addresses to a few persons who they felt confident to contact in order to discuss matters within the child health promotion field, independent of organisation or profession. The persons revealed by this means were in the next step contacted by e-mail and asked the same question. By using the snowball technique the e-mail question was in 15 days spread in up to five links starting from the first invited actors. No reminders were sent out.

**Table 1:** Profession and organisation of initial contacts, number of links and contacts in the network analysis

**Informant**	**Profession**	**Organisation**	**Number of links and contacts**
1	Manager, Division of Public Health	Swedish Association of Local Authorities and Regions	5 links
			69 contacts
2	Public health officer, responsible for a national forum for local and regional public health work	Swedish Association of Local Authorities and Regions	4 links
			44 contacts
3	President, National Public Health Committee	Ministry of Health and Social Affairs, the Swedish Government	3 links
			19 contacts
4	Ombudsman	Ombudsman for Children in Sweden	1 link
			14 contacts
5	Chief secretary	Children´s welfare foundation Sweden	1 link
			7 contacts
6	President	Children´s welfare foundation Sweden	No response
7	President	Swedish Association of Public Health Work	No response

Name, sex, profession, work organisation and geographical location, and contacts between different actors were registered in Excel and analysed in Pajek [19].

Ethical approval was not required for this study. According to the Swedish regulation ethical approval is decreed in research studies where physical procedures are made or sensitive information on individuals is handled [20]. The data collection is this study was made from professionals working in governmental organisations, health care administrations and municipal administrations and did not comprise any ethically sensitive subjects.

## Results

In order to identify influential persons with high credibility in the field of child health promotion, seven leading actors, mainly policy makers, at the national level within the Swedish public health sector were contacted by e-mail. Five of the seven initially contacted persons responded to the e-mailed enquiry. Contact 1 resulted in five response links with a total of 69 contacts. Contact 2 resulted in four response links with a total of 44 contacts. Contact 3 resulted in three response links with in total 19 contacts. Finally, the contacts 4 and 5 resulted in one response link each with a total of 14 and 7 contacts respectively. In Table 1 number of links and contacts emanating from each “start person” are shown.

Forty-four per cent of the informants referred to an actor in the same city or region and 49 per cent to an actor within the same organisational field. Researchers (mentioned by 35 persons), persons working in the public health sector, for example the Swedish National Institute of Public Health or other public health organisations (mentioned by 26 persons) and paediatricians (mentioned by 23 persons) were of most frequent occurrence. The combination paediatrician and researcher was mentioned by 15 persons. Thus, the most often mentioned actors in this network analysis are researchers, public health officials and paediatricians. Four single individuals were mentioned by five to seven other persons. Among these four, three held the combined position paediatrician and researcher, and one was a public official leading a nationwide network regarding children’s health and welfare.

## Discussion

The most often mentioned actors in this network study were researchers, public health officials and paediatricians, or a combination of these professions. Four single individuals were mentioned by five to seven other persons in the network. These four individuals apparently possessed qualities that made several other professionals within the public health sector listen to and trust them. They are not, however, proved to be genuine opinion leaders, matching Roger’s requirements; greater exposure to mass media, greater contact with change agents, greater social participation, more cosmopolite, more innovative and with higher socioeconomic status compared to their “followers” [2]. But they do in fact seem to be persons with high credibility [3], who other professionals feel confident to contact, thus *potential* opinion leaders.

The frequent mentioning of actors holding the combination paediatrician-researcher was to some extent surprising since in Sweden most of the universal public health work is offered by the municipalities [21], for example in preschools, schools and other local environments where children and adolescents spend much time. We hypothesized actors working in the public health sector, for example the National Institute of Public Health or other public health organisations at the local, regional or national level to be of more importance than other groups, which was however not the case. This result may show that the trustworthiness of researchers and paediatricians is high among public health professionals in Sweden.

The informant method and the snowball method were by the authors assessed to be appropriate and low resource-demanding methods to identify *potential* opinion leaders. The snowball method is, according to Valente and Pumpuang, likely to identify leaders to whom others look for advice [11].

The choice of the first contacted individuals, the broad formulation of the e-mail message and the short response time might have influenced the result. We selected seven “start actors” from different arenas and of different professions, mainly policy makers. None of the first contacted individuals were a researcher or a paediatrician. We asked, in the e-mail message, for persons who the respondents felt confident to contact in order to discuss matters within the child health promotion field, thus a very broad question. We did not ask for “leaders” since our intention was to find persons with high credibility, not head of divisions’ etcetera, in the field of child health promotion. With a more narrow formulation, for example limited in organisation, profession or geography, we might have received another result. We were however, interested in the national level which enforced us to be quite broad, both regarding “start actors” and the question formulation. Finally, a more extended response time, as well as reminders, might have resulted in additional response links and more contacts.

## Conclusions

Social network analysis seemed to be a useful method to map influential persons with high credibility, i.e. *potential* opinion leaders, in the national arena of child health promotion in Sweden.

Previously, effectiveness studies of opinion leaders have been performed mostly in the health care sector [4]. We suggest effectiveness studies of opinion leaders to be performed also in the public health sector. When opinion leaders are identified individual visits and other directed measures could be carried out in order to spread relevant knowledge about for example new health promoting methods. This may enable public health actors at the local, regional and national level to more rapidly progress innovations into everyday practice.

## Competing interests

The authors declare that they have no competing interests.

## Authors' contributions

KG and SB designed the study. KG collected and structured the data. MKN performed the network analysis. All authors were involved in drafting and revising the manuscript.
